# Longitudinal metabarcode analysis of karst bacterioplankton microbiomes provide evidence of epikarst to cave transport and community succession

**DOI:** 10.7717/peerj.10757

**Published:** 2021-03-08

**Authors:** Kendall V. Morse, Dylan R. Richardson, Teresa L. Brown, Robert D. Vangundy, Aubrey Bruce Cahoon

**Affiliations:** University of Virginia’s College at Wise, Wise, VA, USA

**Keywords:** Karst cave, Microbiome, Metabarcoding, Community succession, Epikarst

## Abstract

Caves are often assumed to be static environments separated from weather changes experienced on the surface. The high humidity and stability of these subterranean environments make them attractive to many different organisms including microbes such as bacteria and protists. Cave waters generally originate from the surface, may be filtered by overlying soils, can accumulate in interstitial epikarst zones underground, and emerge in caves as streams, pools and droplets on speleothems. Water movement is the primary architect of karst caves, and depending on the hydrologic connectivity between surface and subsurface, is the most likely medium for the introduction of microbes to caves. Recently published metabarcoding surveys of karst cave soils and speleothems have suggested that the vast majority of bacteria residing in these habitats do not occur on the surface, calling into question the role of microbial transport by surface waters. The purpose of this study was to use metabarcoding to monitor the aquatic prokaryotic microbiome of a cave for 1 year, conduct longitudinal analyses of the cave’s aquatic bacterioplankton, and compare it to nearby surface water. Water samples were collected from two locations inside Panel Cave in Natural Tunnel State Park in Duffield, VA and two locations outside of the cave. Of the two cave locations, one was fed by groundwater and drip water and the other by infiltrating surface water. A total of 1,854 distinct prokaryotic ASVs were detected from cave samples and 245 (13.1%) were not found in surface samples. PCo analysis demonstrated a marginal delineation between two cave sample sites and between cave and surface microbiomes suggesting the aquatic bacterioplankton in a karst cave is much more similar to surface microbes than reported from speleothems and soils. Most surprisingly, there was a cave microbe population and diversity bloom in the fall months whereas biodiversity remained relatively steady on the surface. The cave microbiome was more similar to the surface before the bloom than during and afterwards. This event demonstrates that large influxes of bacteria and particulate organic matter can enter the cave from either the surface or interstitial zones and the divergence of the cave microbiome from the surface demonstrates movement of microbes from the epikarst zones into the cave.

## Introduction

Caves form in humid regions underlain by soluble rocks, such as carbonates or evaporites, due to dissolution by circulating meteoric waters acidified by carbonic acid (Klimchouk & Ford, 2000). In mountain belts such as the Appalachians, the development of cave passages is often controlled by pre-existing weaknesses in the bedrock associated with tectonic structures such as fractures, faults, and folds. Epigenic recharge occurs when precipitation and surface streams are intercepted and funneled into the subsurface through highly permeable networks of interconnected conduits in the uppermost bedrock, a zone known as the epikarst. The spatially heterogeneous epikarst can rapidly transmit water though the vadose zone via vertical shafts, and store water and nutrients in soil-filled fissures and voids (Palmer, 2009; Jones, 2013). Perched aquifers may exist in the epikarst due to the lack of drains, and the large porosity contrast with the cave below (Klimchouk, 2000), but do not represent the local or regional water table.

Caves have relatively stable internal atmospheres that have been studied for the purpose of understanding speleothem paleoclimatology (Spötl, Fairchild & Tooth, 2005; Banner et al., 2007; Baldini et al., 2008; Fairchild & Treble, 2009; Ridley et al., 2015; Hasegawa et al., 2018; Kelley et al., 2019), habitat and natural resource conservation (Martin et al., 2010; Jernigan & Carson, 2011; Šebela & Turk, 2011), and speleogenesis and exploration (Dublyanski & Dublyanski, 2000; Wefer & Lucas, 2015). Very high-resolution measurements of meteorological parameters such as temperature, humidity, pressure, and airflow, however, indicate that caves contain distinct microclimates that are temporally and spatially influenced by water and air flux from the surface (Badino, 2010), potentially contributing to the creation of niche habitats. Some common conditions of cave environments are that they are aphotic, except near an entrance, skylight, or artificial light in show-caves, so photosynthesis generally does not occur. These are nutrient-deficient ecosystems with low biomass, in which inhabitants require specific adaptations to low energy environments. Caves support a variety of aquatic and terrestrial habitats, including those associated with streams, pools, sediments, speleothems, crevices, and other surfaces occupied by microbial life as well as micro- and macroinvertebrates (Hobbs & Culver, 2009). The epikarst also supports a distinct faunal community that can be vertically transported into caves by drip waters (Pipan & Culver, 2005).

Oligotrophic cave microbes have been of interest since the 1940s when researchers began culturing them from subterranean environments (Höeg, 1946; Caumartin, 1963; Barton & Northup, 2007). They remain a topic of research due to their roles as primary producers in these extreme environments, their contribution to the formation of cave features, as reservoirs of animal pathogens, as the sources of potentially novel enzymes or metabolites that could be useful in medicine or biotechnology, and as a source of novel microbial mechanisms of antibiotic resistance (Nakaew, Pathom-aree & Lumyong, 2009; Yücel & Yamaç, 2010; Igreja, 2011; Bullar et al., 2012; Dapkevicius, 2013; Wynne & Wang, 2013; Perry, Waglechner & Wright, 2016; Pawlowski et al., 2016, 2018; Adam et al., 2018; Rangseekaew & Pathom-Aree, 2019). Surveys using a range of techniques have shown that prokaryotic microbes ubiquitously inhabit every cave biome, no matter how extreme (Chelius & Moore, 2004; Tomczyk-Żak & Zielenkiewicz, 2016; D’Angeli et al., 2019; Burow et al., 2019).

Initially, cave microbe identification was limited to those that could be cultured and it was suspected that a multitude of microbes with unusual nutrition requirements remained undiscovered (Barton & Northup, 2007). To overcome these limitations culture-independent DNA-based strategies are being used to survey cave microbes with increasing frequency (reviewed in Cheeptham (2013)). One of these, metabarcoding, uses the V4 region of the 16S rRNA gene to not only estimate microbial biomass but also identify microbial species. When combined with deep-sequencing technologies, metabarcoding gives researchers the ability to screen millions of individual DNAs and identify thousands of unique sequences from an environmental sample (Handelsman, 2004; Wooley, Godzik & Friedberg, 2010). To date, this technique has been used to survey microbes in cave soils, speleothems, water, and biofilms in temperate and extreme environments (Ortiz et al., 2013; Wu et al., 2015; Lavoie et al., 2017; Pfendler et al., 2018; Wisechart et al., 2018, 2019; Thompson et al., 2019; Burow et al., 2019; D’Angeli et al., 2019; Zhu et al., 2019; Dong et al., 2020).

In certain groundwater settings such as deep karst aquifers and cave pools with long water residence times (months to years), distinct microbiomes have been described and identified as autochthonous microbial endokarst communities (AMEC). Although AMEC are not specifically defined, they are described as stable aquatic bacterial communities (biofilm and planktonic) comprised predominantly of the Phyla Acidobacteria, Nitrospira, and (γ-, ∆-) Proteobacteria (Farnleitner et al., 2005; Pronk, Goldscheider & Zopfi, 2008). Presumably, most bacteria (including possible AMEC) enter caves through the movement of meteoric water into the subsurface environment, that is, a combination of stochastic flooding and/or sustained seepage that can present itself as droplets on cave formations or create pools fed by groundwater (Laiz et al., 1999; Simon, Benfield & Macko, 2003; Engel & Northup, 2008; Shabarova, Widmer & Pernthaler, 2013; Wu et al., 2015). The newly introduced microbes diverged genetically with time and physical isolation to become unique taxa. In a recent study, Brannen-Donnelly & Engel (2015) analyzed bacterial diversity and community assembly from water, sediments, and mesocosms to identify AMEC in a hydrologically dynamic Appalachian karst cave stream. Each medium supported distinct communities, and several were quite stable, but clear evidence for AMEC was lacking over the duration of the 6-month investigation (Brannen-Donnelly & Engel, 2015). In contrast, meta-barcoding studies have shown that the majority of the microbes on rock walls and speleothems are not found on the surface and may form cave endemic communities (Ortiz et al., 2013; Wu et al., 2015; Lavoie et al., 2017; Thompson et al., 2019). They also show that rock walls and speleothems have distinct individualized microbiomes dominated by a few phylotypes regardless of their proximity to one another. Similarly, cave soil and sediments harbor large numbers of bacteria that were not found in nearby surface soils (Wu et al., 2015; Wisechart et al., 2018; Thompson et al., 2019). Despite the implied importance of water in transporting microbes into cave environments there are few published large-scale surveys of cave aquatic bacterioplankton and even fewer with a temporal component. This study was conducted to apply metabarcoding to water samples collected monthly from a single cave over the course of a year. In doing so we intended to address the following questions: (1) How similar is the bacterioplankton microbiome of cave interior water compared to that of surface water? (2) Does the cave aquatic bacterioplankton microbiome change over time? We hypothesized that the cave bacterioplankton would differ from the surface and that temporal changes in the cave would be minor.

## Materials and Methods

### Panel cave geology and hydrology

**Figure 1:**
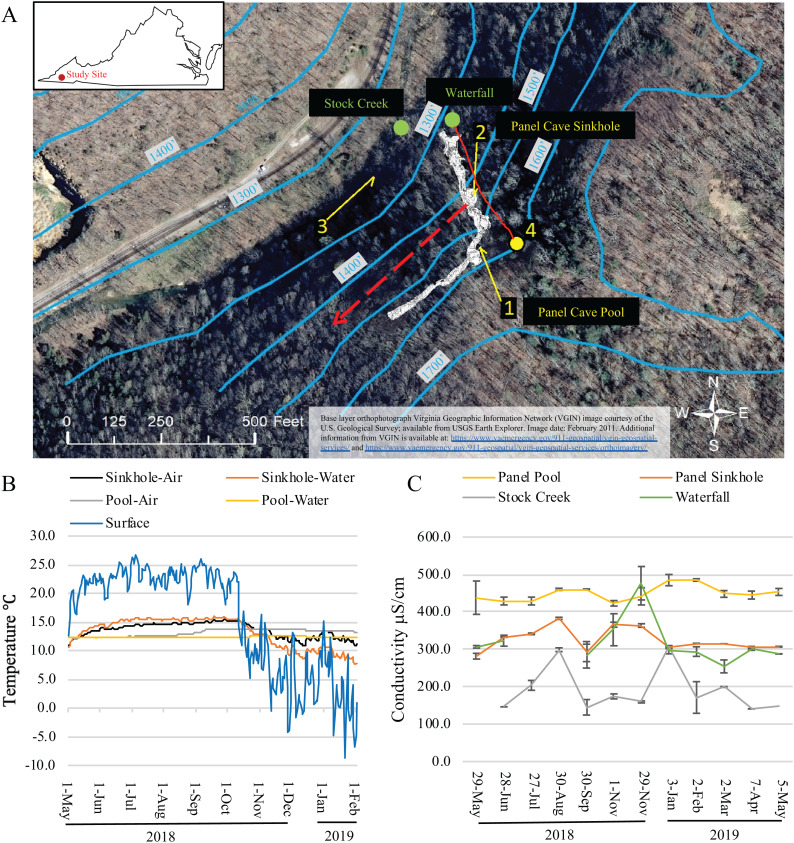
Dye trace, temperature and conductivity of Panel Cave. (A) Study site. Location of dye recovery package locations (1–3) and dye injection site (4) (1 foot = 0.3048 m). The metagenomic surface water sampling locations are labeled with green dots and text. Solid red line indicates flow path of water from surface stream into cave drainage and to sinkhole. Dashed red line shows inferred drainage into Stock Creek based on previous dye tracing. Surface elevation contours are in blue. Base layer orthophotograph Virginia Geographic Information Network (VGIN) image courtesy of the U.S. Geological Survey; available from USGS Earth Explorer. Image date: February 2011. Additional information from VGIN is available at: https://www.vaemergency.gov/911-geospatial/vgin-geospatial-services/ and https://www.vaemergency.gov/911-geospatial/vgin-geospatial-services/orthoimagery/. (B) Daily average air and water temperatures of the two cave sample sites (Sinkhole and Pool) as collected by Onset Hobo H21 data loggers and the daily average air temperature of the surface as collected by an Onset Hobo U23 Pro v2. Instrumentation failure resulted in the loss of data collected from February 2 2019 until the end of the project on May 5, 2019. (C) Water conductivity measurements taken at the surface and cave sites at the times collections were made.

Panel Cave is located within Natural Tunnel State Park in Scott County, Virginia, USA (Fig. 1A; Fig. S1). Permission to perform this research and collect samples was granted by the Commonwealth of Virginia Department of Conservation and Recreation (Research and Collecting Permit NT-RCP-121819). Stock Creek flows by the cave entrance (~395 m asl) at the base of Chestnut Ridge which is underlain by the dolomitic middle Ordovician Chepultepec formation (Miller & Brosge, 1954). These rocks were uplifted and thrust to the northwest along the nearby Glenita Fault during the Alleghenian Orogeny (Brent, 1963), and are highly fractured as a result. As previously described by Thompson et al. (2019), a major feature of Panel Cave is a small stream fed by water spraying from a fissure in the 12 m high ceiling of a large room. The water pools to a depth of 10–20 cm and flows through a narrow channel carved into thick sandy sediments before exiting through a swallet. Known as the “Sinkhole”, this swallet is the deepest point in the cave and is located approximately 55 m from the entrance. A previous dye tracer test confirmed that the cave stream discharges into Stock Creek approximately 1,500 m south of the study site by way of an unmapped spring resurgence. The “Pool” is a still, clear body of water located in a room highly decorated with speleothems in the upper levels of the cave (approximately 100 m from the entrance). The Pool appears to be sourced from condensation and vadose drip water and has no obvious outflow.

The hypothesized contribution of a small sinking stream on Chestnut Ridge, as well as the epikarst, to the hydrology of Panel Cave was determined through two separate dye tracer tests. First, the presence or absence of background fluorescence that could interfere with dye detection was assessed using activated charcoal dye absorption packets (passive samplers) collected from the Pool, the Sinkhole, and Stock Creek (Fig. 1). These were replaced with fresh passive samplers prior to each dye trace. In the first test, 50 gm of Rhodamine (Thermo-Fisher, Asheville, NC, USA) was injected into the stream valley overlying the cave on September 2, 2018. In a second test, 50g of Uranine (Thermo-Fisher, Asheville, NC, USA) was flushed into the septic system of cabins located higher on the ridge to the east of the cave on September 4, 2018. Passive samplers were eluted according to Aley (2019) and analyzed at East Tennessee State University’s Analytical Services Laboratory (Johnson City, TN, USA) using a Jobin Yvon Fluoromax-3 spectrofluorometer.

### Microbial sample collection, DNA extraction, PCR, and sequencing

Water samples were collected from the Panel Cave Sinkhole stream and the Pool (Fig. 1A; Fig. S1). Surface water was collected from two bodies of water: Stock Creek and from the cliff side next to the entrance of the cave which was termed the Waterfall. Both sites were described in Cahoon et al. (2018). Three separate liters of water were collected from each site on each date and treated as independent replicates for the remainder of the processing and analyses. Water was not collected every month from the Waterfall due to extremely low flow. Negative controls were collected by exposing distilled water to the atmosphere at each sampling site. Lab water controls were collected by filling 1 L containers with distilled water in the lab where DNA extractions were to be performed and immediately filtering them.

Water samples were transported to a laboratory at the University of Virginia’s College at Wise and passed through 0.45 μm membranes using a disposable microfunnel with filter (Daigger & Co., Vernon Hills, IL, USA) within 24 h of collection. Filters were stored at −20 °C until DNA could be extracted with Qiagen’s (Germantown, MD, USA) DNeasy PowerWater Kit using the manufacturer’s instructions. These samples were stored at −20 °C and transported to Wright Labs, LLC (Huntingdon, PA, USA) where V4 16S barcodes from the 144 samples plus controls were PCR amplified using the protocol of Caparosa et al. (2011) and TaKaRa ExTaq DNA polymerase (Mountain View, CA, USA). Paired-end reads of 150 bases were produced using Illumina MiSeq technology (reagents kits 3). All raw sequence data were deposited into the National Center for Biotechnology Information USA, sequence read archive, accession PRJNA656557.

All analyses outlined in this paragraph were performed using the QIIME2 platform (Bolyen et al., 2019, v. 2020-8). Primer sequences were removed from both forward and reverse sequences. Paired sequences were filtered, trimmed, dereplicated, merged, and chimeras removed to produce Amplicon Sequence Variants (ASVs) using DADA2 (Callahan et al., 2016). Read counts were normalized by rarefaction (Weiss et al., 2017) at a depth of 100 to accommodate the low counts retrieved from most cave samples. Phylogenies for use in α- and β-diversity tests were constructed using q2-feature-classifier (Bokulich et al., 2018) and the GreenGenes taxonomic training set, gg_13_8_train_set_97 (https://greengenes.secondgenome.com). Although the GreenGenes database has not been updated since 2012 we chose to use it since it had been used in a previous study of Panel Cave (Thompson et al., 2019) and also we found that very few reads were unidentified to the Class level using this database. Pair-wise PERMANOVA scores for beta analysis (Table 1) were generated as part of the unweighted UniFrac analyses. Faith (1992) phylogenetic biodiversity was used to estimate α-diversity for each sample from each site. Beta-diversity comparisons were conducted using unweighted UniFrac comparisons (Lozupone & Knight, 2005) and visualized using Emperor (Vazquez-Baeza et al., 2013). Longitudinal changes were calculated using q2-longitudinal (Bokulich et al., 2018). Shannon’s entropy was used for α-diversity and unweighted UniFrac analyses for β-diversity; both were visualized using volatility plots.

**Table 1:** Non-parametric analysis of variance (PERMANOVA) pair-wise comparisons of UNIFRAC distances from all replicates for each collection site and all collection times.

	Panel Cave—Sink Hole	Panel Cave—Pool	Surface—Stock Creek	Cave
Panel Cave—Pool	2.32 (0.001)	–	–	–
Surface—Stock Creek	5.98 (0.001)	3.34 (0.001)	–	–
Surface—Waterfall	3.52 (0.001)	2.02 (0.001)	2.93 (0.001)	–
Surface	–	–	–	4.27 (0.001)

**Note:**

Individual ASVs and read counts from each set of samples were rarefied (depth 100) and beta-diversity measured using unweighted UniFrac analysis. UniFrac distances were compared using PERMANOVA. *p*-values are in parentheses.

Taxa (Tables S1, S2 and S3) were curated from lists generated from DADA2 (v1.16) as a standalone program in R (Oksanen et al., 2018; R Core Team, 2018, v. 2020-06-22) and the assignTaxonomy function using the GreenGenes taxonomic training set, gg_13_8_train_set_97.

PRIMER-E with the PERMANOVA add-on (Clarke & Gorley, 2015) was used to estimate the contribution environmental factors made to changes in the microbiome. Differing read counts for each taxa were normalized using presence/absence and a distance matrix produced using Bray-Curtis divergence. Environmental variables (water temperature, dissolved oxygen, pH, conductivity, chlorine, nitrate, and ammonia levels) were normalized using the square-root function and a resemblance matrix generated using Euclidean distance. Correlations between environmental factors and microbiomes were calculated using BEST, and dbRDA analyses.

### Meteorology and other environmental measurements

Onset (Bourne, MA, USA) Hobo H21 data loggers with sensors to measure air temperature, relative humidity, air pressure, soil moisture, water temperature and light level were installed in two locations within the cave. One was located within the Sinkhole stream just before it exited via the swallet, and the second data logger was located in the room containing the Pool, roughly 100 m from the entrance. Surface temperature and relative humidity near the entrance to the cave were measured with an Onset Hobo U23 Pro v2. Temperature and precipitation data from the National Oceanic and Atmospheric Administration’s Weather Observer Station in Wise, VA and the Prism Climate Group, Northwest Alliance for Computational Science & Engineering (https://prism.oregonstate.edu) were used to supplement external measurements. Water temperature, pH, Specific Conductivity, dissolved oxygen, chloride, nitrate and ammonia were also measured using a Vernier LabQuest 2 meter in water samples collected monthly.

## Results

### Dye-trace, and meteorology of two sites in panel cave

The dye tracer tests confirmed a direct hydrologic connection between a sinking stream on the surface of Chestnut Ridge and Panel Cave’s Sinkhole stream, and a much weaker connection between a ridgetop sewage drain field and the cave stream. Fluorescence spectra for the Pool was dominated by the signature of dissolved organic matter with no clear evidence of the presence of either dye (Coble et al., 1990; Hernes et al., 2009; Aley, 2019).

Average daily surface air temperatures ranged from a high of 26.7 °C to a low of −8.7 °C over the course of this study. Minimum external air temperatures dropped steadily throughout October coinciding with annual autumnal leaf senescence (Fig. 1B). Temperatures in Panel Cave had a much narrower range but differed between the two sites (Fig. 1B). Daily averages of the Panel Sinkhole had air temperatures ranging from 11.0 to 14.5 °C and average daily water temperatures ranging from 8.3 to 15.3 °C. Panel Pool experienced a dampened response to changes of outside air temperatures with daily average air temperatures ranging from 12.2 to 13.7 °C and daily average water temperatures from 12.2 to 12.5 °C.

The conductivity of the four sample sites was most similar between the Waterfall and Sinkhole (Fig. 1C). Stock Creek had the highest standard deviation in conductivity and temperature while the Pool consistently had the highest conductivity with the least variance, except for May 2018 sample.

### Cave microbial taxa and interior vs exterior comparison

Over the course of 12 months, 144 samples were collected from four sites (two cave interior and two surface, plus field and lab controls). Of those, 106 cave and surface samples were successfully metabarcoded (Panel SinkHole *n* = 24; Panel Pool *n* = 16; Stock Creek *n* = 35; Waterfall *n* = 28; reasons for failures are discussed below), yielding 4,413,398 sequences. After quality control and read pairing were completed 2,367,204 reads remained. From those, 1,854 putative prokaryotic ASVs were identified. All ASV taxa and raw sequence counts are listed in Tables S1, S2, and S3.

**Figure 2:**
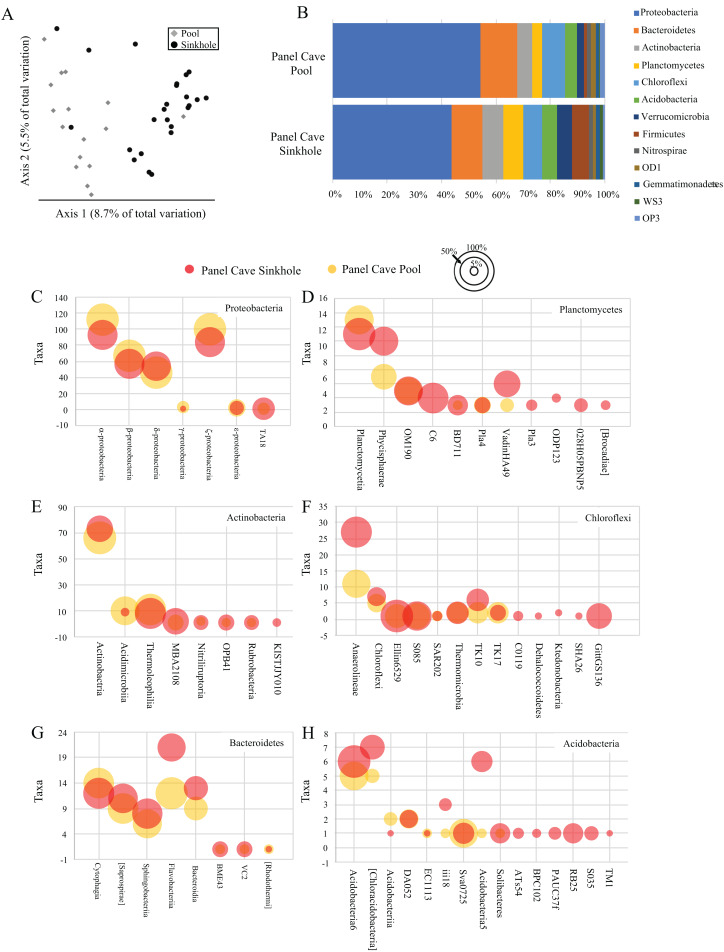
Panel Cave bacterioplankton microbiomes. (A) PCoA plot of samples collected from two sites within Panel Cave, the Pool (diamonds) and Sinkhole (circles). Plot was generated from rarefied data (depth = 100) and unweighted UniFrac distances. (B) The relative abundance of taxa for the most abundant phyla found in the two cave sites across all time points, that is, => 1% of the total number of instances taxa from that phyla occurred in a sample. (C–H) Bubble plots demonstrating the total number of taxa (*y*-axis) from each Class within the six most abundant phyla; C—Proteobacteria, D—Planctomycetes, E—Actinobacteria, F—Chloroflexi, G—Bacteroidetes, and H—Acidobacteria. Bubble sizes represent the percentage of samples collected from each site, containing representatives from that Class from the Panel Cave Pool (yellow) and Panel Cave Sinkhole (red).

A comparison of all the samples collected over the course of the year from the two cave sites by principle coordinate analysis (PCoA) suggests each body of water had a moderately dissimilar microbiome (Fig. 2A). A Kruskal–Wallis test of the alpha-diversity of each body of water suggested differences were insignificant (Table S4), and a PERMANOVA test of beta-diversity suggested a low yet statistically significant dissimilarity between the two sites (Table 1). Overall, 24 phyla and 21 candidate divisions were detected in the water collected from the cave sites and the 15 most abundant of these comprised ~96% of the total microbiome (Table 2; Table S5). The most abundant taxa inside Panel Cave occurred in the phylum Proteobacteria, whose ASVs comprised 21.1% and 24.6% of the total number recovered from the Sinkhole and Pool sites, respectively (Fig. 2B and Table 2). The most abundant Proteobacterial classes were, in decreasing abundance, α-, ζ, β, and δ-proteobacteria (Fig. 2C; Table S6). The other major phyla, defined as comprising >5% of the taxa of one or both of the cave sample sites were Bacteroidetes, Actinobacteria, and Planctomycetes, Chloroflexi, and Acidobacteria (Fig. 2B; Table S5). Within these phyla there are some notable differences between the two sample sites (Figs. 2C–2H; Table S6). For example, within the Chloroflexi there were many more taxa from the class Anaerolineae (27) in the Sinkhole than in the Pool (11), and taxa from five classes found in the Sinkhole were not detected in the pool. In Bacteroidetes the taxa richness of the class Flavobacteria was higher in the Sinkhole (21) than in the Pool (12). Post-hoc Wilcoxon signed-rank tests comparing the abundance of each phylum and each class at each site was performed and there were no statistically significant differences at these taxonomic levels for the two cave sites.

**Table 2:** Relative amounts (%) of the most abundant prokaryotic phyla found in water collected from each.

Taxonomy	Panel Cave—Sinkhole	Panel Cave—Pool	Surface—Stock Creek	Surface—Waterfall
Phylum				
Proteobacteria	21.1298	24.6314	24.6149	13.6782
Actinobacteria	5.3823	6.2668	6.2626	6.7682
Planctomycetes	3.8566	2.4424	2.4287	3.2955
Chloroflexi	3.4225	1.6429	1.6499	4.8193
Bacteroidetes	3.3458	3.8238	3.8178	6.6266
Acidobacteria	2.6616	1.9437	1.9424	3.4727
Verrucomicrobia	2.6482	1.1394	1.1386	
Firmicutes	2.9777	0.5269	0.5234	0.6733
Nitrospirae	0.7469	0.6367	0.6363	0.7872
OD1	0.5568	0.9718	0.9712	0.7796
Unassigned	0.2852	0.1005	0.7368	1.0276

**Figure 3:**
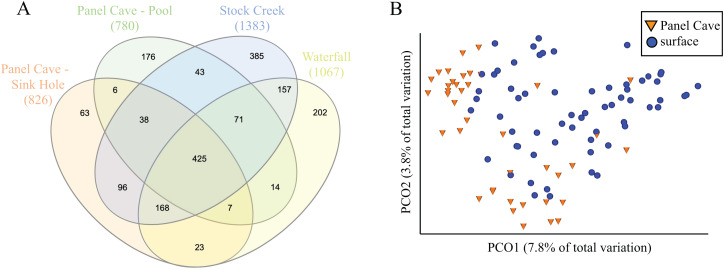
Comparison of bacterioplankton in Panel Cave to the surface. (A) Venn diagram representing ASV taxa unique to each sample site and those found at more than one. (B) PCoA plot of samples collected from Panel Cave and surface sites. Plot was generated from rarefied data (depth = 100) and unweighted UniFrac distances.

A comparison of the taxa found at all four sites, cave and surface, is presented in Fig. 3. Of the 1854 ASVs, 245 (13.1%) were only found in water collected from Panel Cave. Most of these cave specific ASVs (173/245, 70.6%) were identified from the epikarst-fed Pool sample site. A total of 744 (39.7%) of the 1854 ASVs were unique to surface samples and 885 (47.2%) occurred in both cave and surface water. A PCoA comparison of the ASV’s from the cave interior vs surface demonstrated a partial delineation between the cave and surface samples (Fig. 3B). However, the two most abundant PCO dimensions, account for under 13% of the variations between the surface and the cave interior, suggesting multiple variables are responsible for differences between these two environments. A second version of the PCoA plot where each sample site is represented by a unique marker is included as Fig. S2. Comparisons were made for all possible combinations of interior vs surface samples using α-diversity (Table S1) and only two comparisons, Surface Waterfall-Panel Cave Pool and Surface Waterfall-Surface Stock Creek were significantly different according to Kruskal–Wallis tests. β-diversity metrics (unweighted UniFrac) were also compared using PERMANOVA analysis and the Surface Stock Creek—Panel Cave Sinkhole had the largest significant F-ratio suggesting they exhibited the greatest overall difference. Post-hoc Wicoxon signed-rank tests comparing the abundance of each phylum and each class between the cave and surface was performed and there were no significant differences at these taxonomic levels.

### Longitudinal changes in panel cave’s microbiome

**Figure 4:**
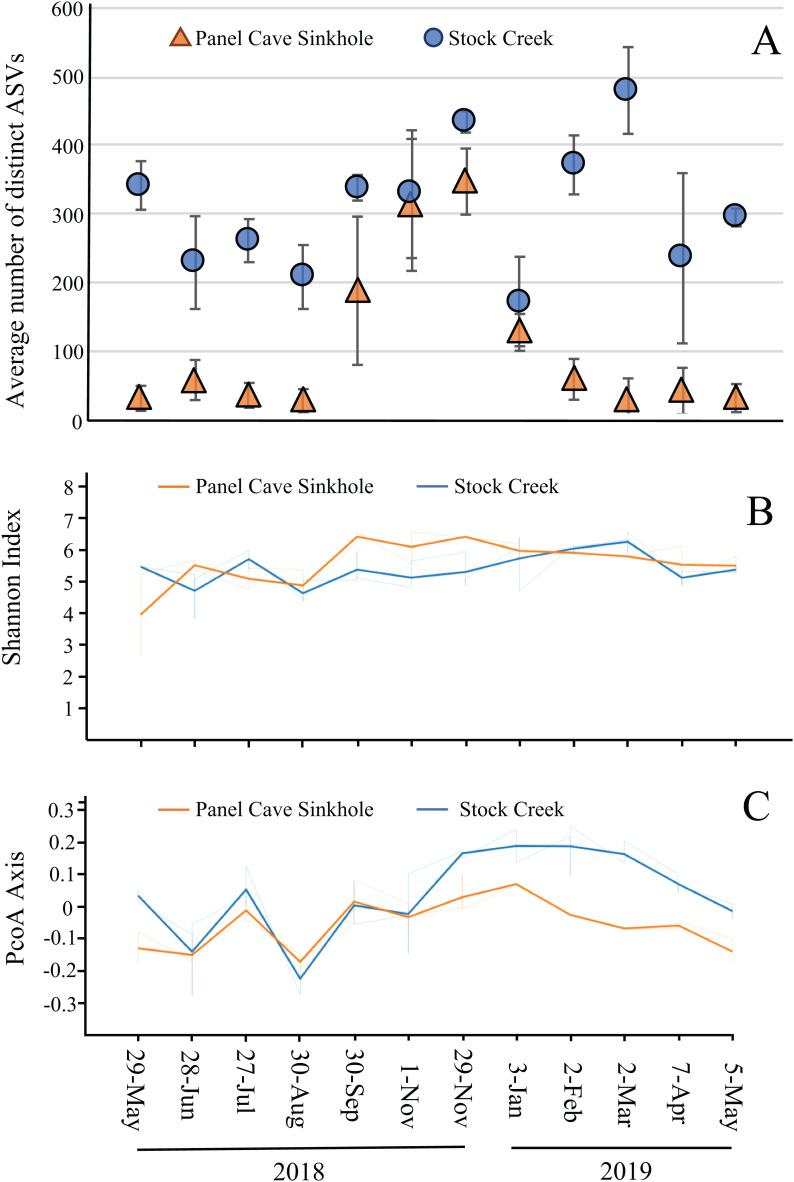
Microbiome longitudinal comparisons of cave and surface water. (A) Average number of distinct ASVs identified at each timepoint (orange—Panel Cave sinkhole, blue—Stock Creek surface site), error bars = SE. (B) Volatility plot based on Shannon indexes (alpha diversity) calculated for all replicates collected at each timepoint (orange—panel sink hole site, blue—stock creek surface site). (C) Volatility plot based on PCoA distances (beta-diversity) calculated for all replicates collected at each timepoint (orange—panel sink hole site, blue—stock creek surface site).

The average number of distinct ASVs found in each sample from the Panel Cave Sinkhole and Stock Creek sites are shown in Fig. 4A. These two sites are highlighted because sequence data was successfully collected for all 12 months and provides the most complete picture of longitudinal change. In the Panel Cave Sinkhole, the average (±SE) number of detectable ASVs was relatively low for most of the year, 59.7 ± 7.5. This increased dramatically, however, beginning in early fall and subsiding in early winter (252 ± 38), peaking in late November (ASV = 347 ± 48). Unlike Panel Cave, the average number of ASVs on the surface was much higher, 316 ± 19.5, but there were changes over the course of the year, such as decreases during late spring and summer.

The observed increase in ASV richness, biodiversity, and read counts in the Panel Cave Sinkhole in the late fall could have been due to the introduction of nutrients and/or an influx of microbes from the surface and/or epikarst. To address this, a comparison of the similarities between the cave and surface samples at each of these time points was performed using PERMANOVA analyses (Table 1). The F-ratio between Panel Sinkhole and Stock Creek was the highest of any of the pairwise comparisons, suggesting the greatest difference. We also generated longitudinal volatility plots to compare the α (Shannon) and β (PCoA) diversities between Panel Sinkhole and Stock Creek at each time point. The Shannon indexes between the two sample sites diverged (albeit insignificantly) during the bloom months suggesting minor changes in the amounts of diversity between the two sites (Fig. 4B). PCoA comparisons demonstrated that late in the bloom (29 November) the microbes present at the two sites diverged significantly and this change persisted for several months (Fig. 4C).

**Figure 5:**
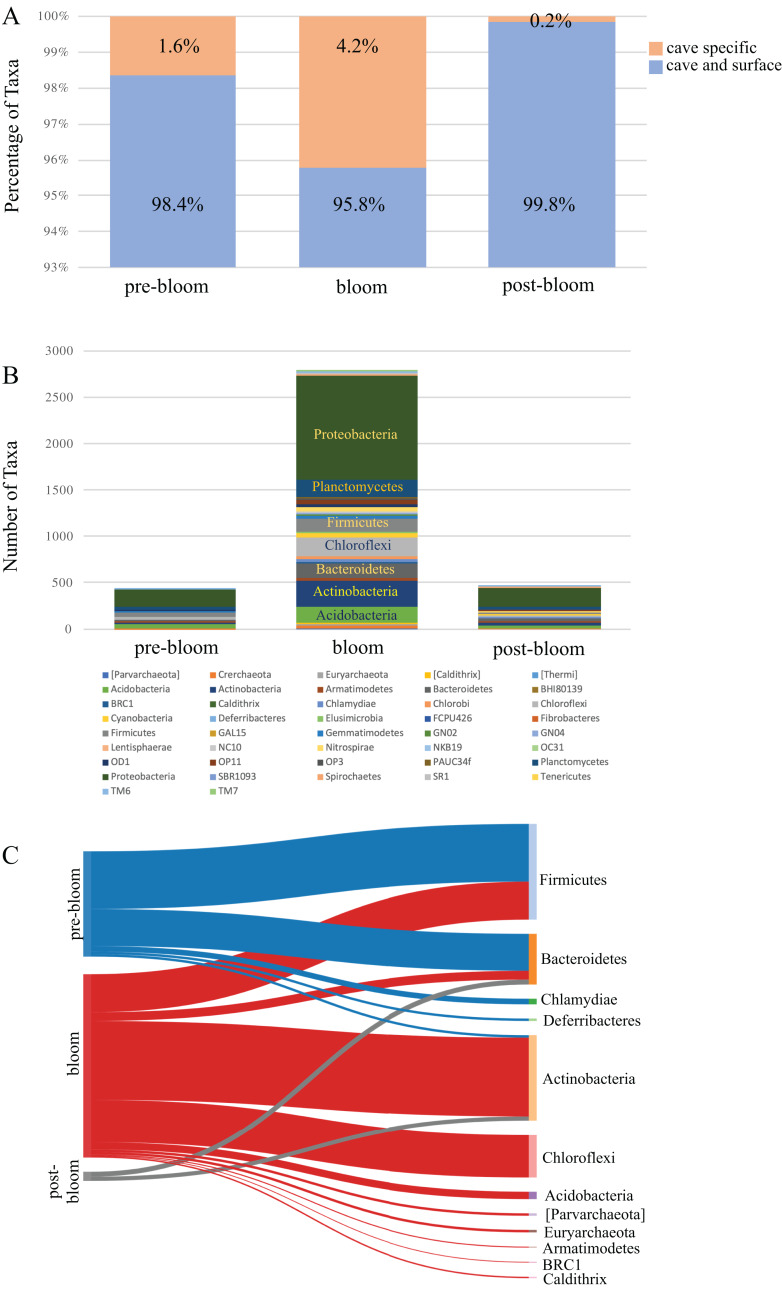
Community succession of the Panel Cave Sinkhole microbiome. (A) Stacked bar plot demonstrating the number of taxa (distinct ASVs) detected from each phylum in the Panel Cave sinkhole before, during, and after the bloom. (B) The percentage of taxa (distinct ASVs) found only in cave water collected from the Panel Cave sinkhole (orange) and those found both in Panel Cave sinkhole and Stock Creek (blue), before, during, and after the bloom event. (C) Sankey diagram showing the relative number of cave specific taxa from each phylum before (pre-), during (bloom), and after (post-) the bloom.

A comparison of the taxa found uniquely in the cave to those shared between the cave and the surface revealed that the proportion of cave-specific taxa increased during the bloom months (Fig. 5A) but decreased dramatically afterwards. Since the number of cave specific taxa was reduced we analyzed the phyla to see which had been affected. The taxa detected from all the cave phyla are presented in Fig. 5B and show a marked increase during the bloom and decrease afterwards, specifically among the Proteobacteria, Actinobacteria, Acidobacteria, and Chloroflexi. A focus on ASV taxa unique to the cave revealed decreases among taxa in the phyla Firmicutes and Bacteroidetes, while Actinobacteria, Chloroflexi, and Acidobacteria increased during the bloom but were once again undetectable after the bloom (Fig. 5C). Interestingly, many of the cave specific microbes detected prior to the bloom were undetectable once the bloom subsided. The most highly affected were taxa from the phyla Firmicutes, Chlamydiae, and Deferribacteres (Fig. 5C).

The sequencing process was unsuccessful for some of the Panel Cave Pool and Waterfall sampling times, so the longitudinal data sets were incomplete. A summary of the average number of unique ASVs from all sites and times (complete and incomplete) are shown in Fig. S3. The Panel Pool (epikarst-fed and very low flow velocity) had a significantly higher overall average number of ASVs, than the pre- and post-bloom Sinkhole (surface fed, constantly running stream) according to a Student’s *t*-test (*p* = 0.017). The surface Waterfall samples had a significantly higher different number of taxa than Stock Creek (*p* = 0.00001). The Waterfall site was dry during the mid-late summer months so no samples could be collected.

### Correlation of environmental factors to longitudinal microbiome changes

**Figure 6:**
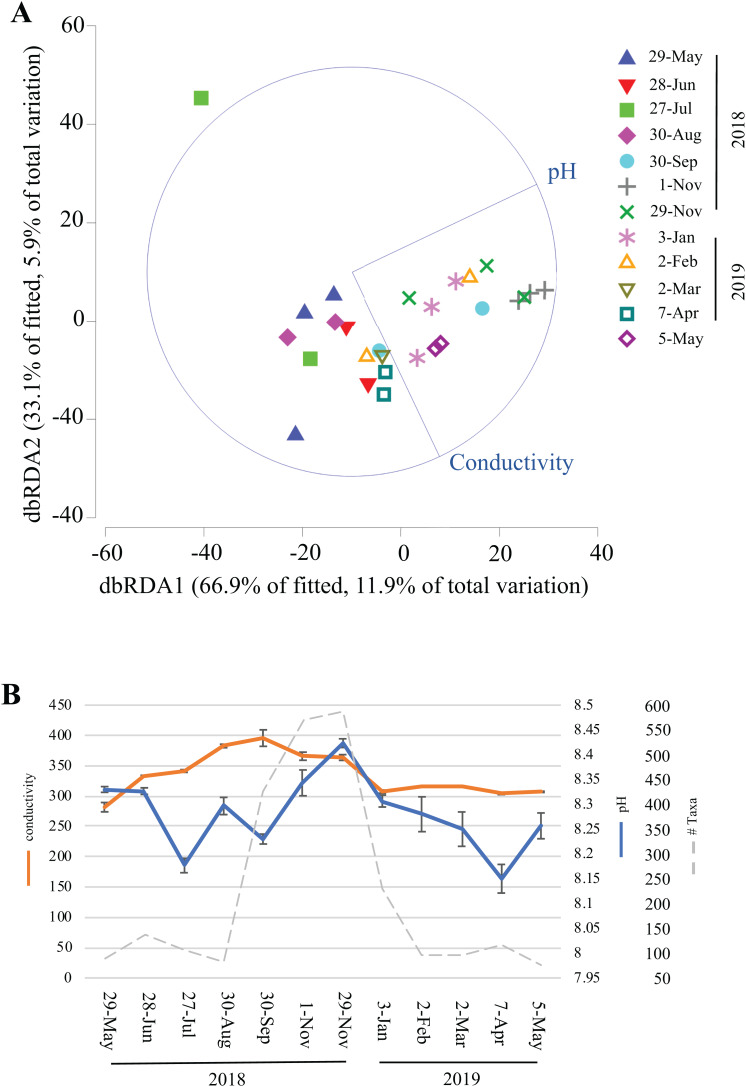
Environmental factors associated with the bacterioplankton bloom in Panel Cave Sinkhole. (A) dbRDA plot of the Panel Cave sinkhole sample distribution related to the two environmental variables that BEST modeling suggested were correlated to changes in the microbiome. (B) Average conductivity and pH values (±SEM) of the Panel Sinkhole water samples along with the total number of reads collected at each time point.

Over the course of this study, environmental data (temperature, humidity, light, dissolved oxygen, conductivity, chlorine, nitrate, and ammonia) were collected inside Panel Cave at the two sample sites. This enabled correlation modeling and analyses to try and determine if the microbial bloom occurred due to changes in the environmental conditions in the cave. First, BEST analysis was conducted, which suggested that changes in pH and conductivity coincided with the change in the microbiome, may explain 31.5% of the variation, and that all other factors combined explained <1% of the variation. A focused analysis on conductivity and pH was then completed using dbRDA (Fig. 6A) and pH aligned more closely with the distribution of the fall bloom samples than conductivity. A line graph with pH, conductivity, and the read counts from the fall microbial bloom suggests that the shift to alkalinity in the Panel Cave Sinkhole coincided with the increase in bacteria (Fig. 6B).

## Discussion

In this study, environmental DNA metabarcoding was used to survey planktonic microbes from cave water collected from two sites within a single cave over the course of a year. Our purpose was to characterize the bacterioplankton micriobiome of a cave whose soils and speleothems had been assayed in a previous study (Thompson et al., 2019). This approach was taken to determine (1) the similarity of the bacterioplankton microbiome between water in the cave interior compared to surface water; and (2) if the cave prokaryotic microbiome changes over time.

### Cave microbial taxa

The microbiomes of the two sample sites had moderately distinct PCoA distributions (Fig. 2A) and a relatively low PERMANOVA F statistic (Table 1) suggesting they were mildly dissimilar. These differences may be related to their contrasting flow regimes. The dye trace experiments demonstrated a presumably direct connection between the surface and the Sinkhole stream but the Pool dye results were negative to ambiguous. Also, the passive sampling strategy used for the dye trace experiments meant we were unable to determine flow through times for the tracer tests, but based on similar dye traces we assume that the flow-through/residence time from sinking stream to the cave is on the order of hours, including residence time flowing across the floors of the big room before entering Sinkhole swallet; whereas, residence time for water in the epikarst-fed such as Pool is likely days to weeks.

The microbiome compositions from two bodies of water from Panel cave is inconsistent with observations from Bärenschacht cave system in Switzerland where community overlap between different freshwater pools was found to be very low (Shabarova & Pernthaler, 2010). It is also inconsistent with speleothem studies that have shown cave features can harbor dissimilar assemblages of microbes regardless of their proximity (Legatzki et al., 2011, 2012; Ortiz et al., 2013; Lavoie et al., 2017; Wu et al., 2015). The most abundant bacterioplankton phyla in Panel cave—Proteobacteria, Bacteroidetes, Actinobacteria, and Planctomycetes, Chloroflexi, and Acidobacteria are common constituents of cave microbiomes (reviewed in Tomczyk-Żak & Zielenkiewicz (2016)) including bacterioplankton in karst (Brannen-Donnelly & Engel, 2015; Shabarova & Pernthaler, 2010) and extreme environments (Burow et al., 2019; D’Angeli et al., 2019).

Most other published metabarcoding studies have surveyed soil and speleothems (Porca et al., 2012; Ortiz et al., 2013; Wu et al., 2015; Mendoza et al., 2016; Lavoie et al., 2017; Vardeh, Woodhouse & Neilan, 2018; Wisechart et al., 2018, 2019; Thompson et al., 2019; Dong et al., 2020). The most abundant phyla identified in these studies were also found in the Panel Cave water samples. Different phyla appear to predominate, however, in the cave soil vs water microbiomes. In soil, Actinobacteria is the most abundant, followed by Proteobacteria (Wu et al., 2015; Wisechart et al., 2018, 2019; Thompson et al., 2019; Zhu et al., 2019). In the water samples collected in Panel Cave, Proteobacteria clearly dominated the microbiome (>21% of the total taxa), with either Bacteroidetes or Actinobacteria as the second most abundant (>5%). This is consistent with the aquatic bacterioplankton of 8 karst caves in the Yunnan-Guizhou Plateau of China (Zhu et al., 2019). Also, Wu et al. (2015) observed the phylum Nitrospira was more prominent inside the Jinjia Cave in the western Loess Plateau of China than in surface soils, which is consistent with our observations from water inside and outside of Panel Cave. Our results, did differ from these other studies in the Order that was most abundant, for example β-Proteobacteria were most abundant in the Bärenschacht whereas α-Proteobacteria were most abundant in Panel Cave.

Differences between the soil, speleothems, and water in Panel Cave occurred among those phyla comprising minor proportions of the microbiome. The proposed divisions AD3 and GOUTA4 detected in soil and on speleothems by Thompson et al. (2019) were not found in the bacterioplankton. Phyla identified from Panel Cave water that were previously unreported comprised minor components—BHI80139, Deferribacteres, Elusimicrobia, FBP, FCPU426, Fusobacteria, GAL15, Lentisphaerae, NKB19, OC31, PAUC34f, SBR1093, Spirochaetes, SR1, Synergistetes, WPS2, Caldithrix, and Kazan3B28.

### Cave vs exterior microbiome comparison

The majority of Panel Cave’s bacterioplankton microbiome, 86.9%, was also found in surface water. This provides a stark contrast to the metabarcoding identified assemblages of cave speleothems and soils compared to the surface. Rock walls and speleothems have distinct microbiomes and share very few microbes with the exterior environment, the highest reported overlap being 21% (Ortiz et al., 2013; Wu et al., 2015; Lavoie et al., 2017; Thompson et al., 2019). Cave soils share more microbes with the surface than speleothems but it is still relatively low, for example, there was a 53.8% overlap between Appalachian karst caves and the surface (Thompson et al., 2019) and comparably low overlaps have been reported from other karst environments such as the Jinjia Cave (Wu et al., 2015) and the Manao-Pee cave in Thailand (Wisechart et al., 2018). Those observations suggest that cave features and soils harbor a greater proportion of cave-specific microbes than cave water. Comparisons of adjacent speleothems and lava cave mats have shown that each has a distinct microbiome regardless of their proximity (Ortiz et al., 2013; Lavoie et al., 2017), which would be the case if microbes are rarely and randomly transported to aerial solid substrates. It has also been observed that bacterial abundance and richness in a karst cave pool can be temporarily increased by a flood event but that richness degrades as the pool stagnates (Shabarova, Widmer & Pernthaler, 2013). So even though bacteria are introduced into caves on a regular basis, the vast majority are unable to survive in the cave environment, even fewer would be transported into the soils or onto speleothems, and then fewer would have the ability to survive on a rock surface. This reinforces that caves provide niche habitats for microbes with highly specialized metabolic adaptations but that they are limited to the walls, speleothems, and soils.

Our survey of bacterioplankton in Panel Cave and the surrounding surface demonstrates that water exchange transports bacteria between the surface and the subsurface, albeit at varying rates depending on conduit vs epikarstic transport. These bacterioplankton, however, must rarely colonize the cave. This is consistent with a study by Brannen-Donnelly & Engel (2015) who demonstrated that transport of microbes from the surface and through an epikarst layer did not result in the formation of AMEC in an Appalachian karst cave stream. We would like to note, however, that the majority of the cave specific ASVs we recovered were found in the Panel Pool, raising the possibility that AMEC may exist in cave water bodies fed by epikarstic water and very low flow rates.

### Longitudinal changes in the planktonic microbiome

Water samples were collected over the course of a year to maximize the survey of biodiversity and to see if changes in the microbiome could be detected. Reads and the number of taxa were low in samples collected from the Panel Cave sites and for some months no reads were detected from the Pool site. We believe this is due to very low numbers of bacteria present in this body of water fed only by groundwater seepage. Most of the water samples removed from the Sinkhole and Pool sites had low species richness and bacterial populations which is consistent with previous studies. Cave interior microbial biomass has been calculated to be 150,000 times lower than on the surface (Barton et al., 2006) and a quantitative PCR approach estimated surface soils to have 1–2 orders of magnitude greater microbial biomass than cave samples (Wu et al., 2015). The number of bacterioplankton present in karst cave pools, as determined by microscopic examination, has been reported to be very low (Shabarova & Pernthaler, 2010) and is lowest in very low velocity bodies of water (Shabarova, Widmer & Pernthaler, 2013), like the Pool site in Panel Cave. In contrast, the Sinkhole site experienced a very obvious change in its bacterioplankton profile with an increase in its species richness and abundance in the late fall and early winter that rivaled the richness and abundance seen on the surface. After this “bloom” event subsided and the levels of bacteria were once again reduced to previous levels, the taxa comprising the microbiome were not the same. A comparison of the Panel Sinkhole to the Surface revealed an increase in diversity in the cave during this event resulting in a microbiome that diverged from the surface before and afterwards (Figs. 4B and 4C). There was also an increase in the number of cave-specific taxa during the bloom. We interpret these results as evidence that perched groundwater (and accompanying bacteria) were transferred from storage in the epikarst rather than directly from the surface.

It is generally believed that microbes are constantly entering caves from meteoric water (Simon, Benfield & Macko, 2003; Engel & Northup, 2008). Three studies have implied the transport of microbes from the surface into cave pools. Laiz et al. (1999) cultured bacteria from water dripping from cave formations and found taxa similar to those in surface soils rather than those on damp cave ceilings. Shabarova, Widmer & Pernthaler (2013) noticed that stochastic flood events brought microbes from other environments into deep alpine karst cave pools but that the species richness and abundance would dramatically decline over time as the pools were starved of fresh recharge. Those pools would experience a nearly complete water replacement by flooding, which prompted the authors to speculate that these systems represented an extreme example of the mass effect theory of bacterial biogeography, that is, changes in a microbiome can be attributed to stochastic dispersal of current microbes (emigration) followed by recruitment of new microbes (immigration) from the displacement event (Logue & Lindström, 2008). Wu et al. (2015) found the elemental and bacterial composition of water from a shallow karst cave to be much more similar to the surface than cave sediment or surfaces of speleothems. They posited that a cave receiving meteoric water would have a constantly changing microbial community due to drip water but the very low abundance and richness was attributable to dispersion of the microbes as the water percolated into the cave through semi-solid substrate. We do not know if the event we observed was due to flooding since we did not install flow meters at any of the sample sites, but it did follow several fall storm events after several months of drought. We also noticed a dark precipitate of unknown composition on the sandy substrate when the collections were made along with a noticeable change in pH and conductivity (Table S7).

The Sinkhole stream is very likely fed by a combination of surface and epikarstic water. With the connection to the surface and epikarst we propose three possible explanations for the bloom and changes in hydrochemistry. (1) The bloom and the hydrochemistry change could be coincidental. (2) The bloom may have changed the pH of the water as the microbes utilized the more labile organic matter introduced from the surface. As respiration increased, CO_2_ would have off-gassed into the water and cave interior increasing the pH and conductivity. (3) The change in pH and bacterial biodiversity were the result of an increase in water flow from the epikarst into the cave. We posit that the evidence supports the third explanation. We hypothesize that the appearance of large numbers of bacteria in Panel Cave was associated with dissolved and particulate organic matter transported into the epikarst from the surface due to plunging temperatures, rain-driven recharge events, and leaf fall. The increases in pH and conductivity may have been due to the movement of calcite-saturated water that was pushed into Panel Cave by a pressure pulse (piston effect) of older epikarstic water pushed through the vadose zone by fresh recharge (Ford & Williams, 1994). The sample area and Chestnut ridge is heavily forested and some of these changes in the fall could also be associated with root respiration shutting down, which would lower P_CO2_ in the soil zone as winter approaches (Shilling & Jacobson, 2015). The increase in cave-specific microbes in the Panel Sinkhole suggests they were not transported directly from the surface but could have been residing in stored epikarst water. The epikarst areas are known to harbor a diverse array of invertebrate fauna (Pipan & Brancelj, 2004; Pipan & Culver, 2005) and would presumably also house microbes. We currently do not know if this exchange of water occurs regularly or was due to a stochastic set of meteorological and geological events. A temporal metabarcoding study by Brannen-Donnelly & Engel (2015) in a comparable Appalachian cave stream in Kentucky saw no significant changes in bacterial richness from June to December, suggesting the later may be true.

## Conclusions

In this study, we used a combination of geological, hydrological, and molecular genetic techniques to conduct a year-long longitudinal planktonic microbiome metabarcoding survey of cave water. Our results demonstrated that an underground constantly flowing body of water (Panel Cave Sinkhole) fed by a combination of surface and epikarst water, transported microbes from the surface and epikarst zone to underground systems. These results demonstrate that waters within karst caves with direct hydrologic connections to the surface lack the endemic populations of cave bacteria found in the soils and features of these same caves. We also witnessed a dramatic change in the underground bacterial population which coincided with a change in pH and an increase in microbes not found on the surface which suggests an influx of microbes from the epikarst zone.

## Supplemental Information

10.7717/peerj.10757/supp-1 Supplemental Information 1 Map of Panel Cave. The two sample sites, sinkhole and pool, are denoted by small red x’s. The sinkhole water originates from a sinking stream on the surface, presumably passes through an epikarstic layer, and enters the cave through a ceiling fissure #35 (locations are marked by circled numbers on map) with no other obvious surface connection (i.e. light penetration). It collects as a broad and shallow body of water and exits through a rock fissure #10 to form a stream that exits by way of a swallet #27. The pool #18 is a very low velocity body of water fed from groundwater seepage.

10.7717/peerj.10757/supp-2 Supplemental Information 2 PCoA plot of samples collected from Panel Cave and surface sites.

10.7717/peerj.10757/supp-3 Supplemental Information 3 Microbiome longitudinal comparisons of all cave and surface water sites. (A) Bubble plot of the average ASV richness and the average total number of prokaryotic reads collected from each site at each timepoint. The y-axis represents the average ASV richness, bubble sizes represent the average number of reads, and the x-axis represents sample times. (B) The average calculated Shannon Indexes with standard errors for each for each site and time.

10.7717/peerj.10757/supp-4 Supplemental Information 4 Summary of all cave taxa identified by this study.

10.7717/peerj.10757/supp-5 Supplemental Information 5 Summary of taxa only found in Panel Cave during this study.

10.7717/peerj.10757/supp-6 Supplemental Information 6 Summary of taxa found in surface water during this study.

10.7717/peerj.10757/supp-7 Supplemental Information 7 Pairwise Kruskall-Wallis comparisons of each site based on Faith’s phylogenetic diversity scores for each replicate.

10.7717/peerj.10757/supp-8 Supplemental Information 8 Microbial phyla found over the course of this study.

10.7717/peerj.10757/supp-9 Supplemental Information 9 Microbial classes found over the course of this study.

10.7717/peerj.10757/supp-10 Supplemental Information 10 Environmental data collected at the four sample sites.

10.7717/peerj.10757/supp-11 Supplemental Information 11 Environmental raw data.

## Additional Information and Declarations

### Field Study Permissions

The following information was supplied relating to field study approvals (i.e., approving body and any reference numbers): Specimens were collected at the Natural Tunnel State Park in Duffield, VA, USA. Permission was granted by the Commonwealth of Virginia Department of Conservation and Recreation (Research and Collecting Permit NT-RCP-121819).

### Data Availability

The following information was supplied regarding data availability: All raw sequence reads are available at GenBank’s SRA database: PRJNA656557.
