# Supplementary figures and images for: Longitudinal metabarcode analysis of karst bacterioplankton microbiomes provide evidence of epikarst to cave transport and community succession
Source: PeerJ. 2021 Mar 8;9:e10757. doi: 10.7717/peerj.10757 (PMC7950216; doi:10.7717/peerj.10757)

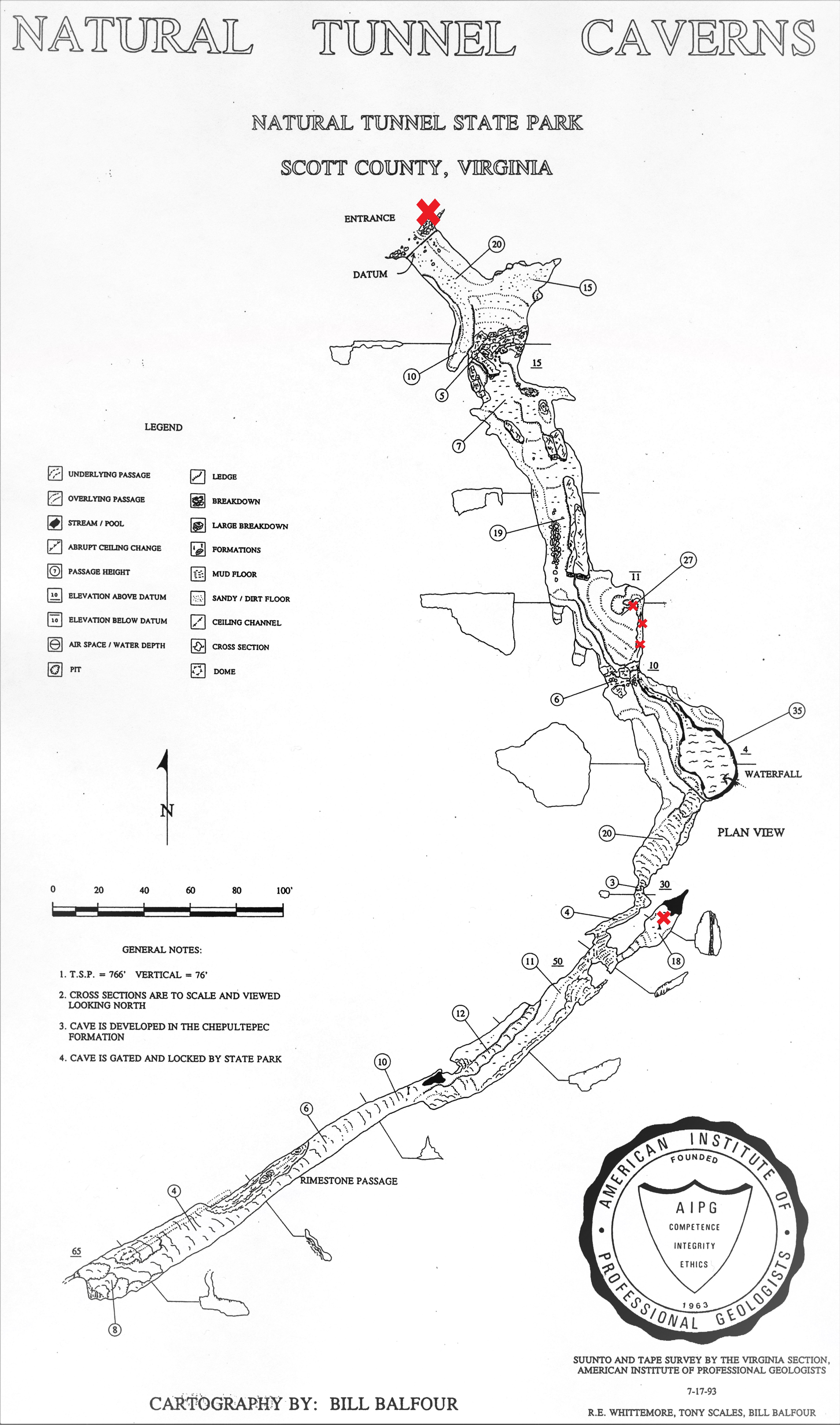

Supplement: Supplemental Information 1 — The two sample sites, sinkhole and pool, are denoted by small red x’s. The sinkhole water originates from a sinking stream on the surface, presumably passes through an epikarstic layer, and enters the cave through a ceiling fissure #35 (locations are marked by circled numbers on map) with no other obvious surface connection (i.e. light penetration). It collects as a broad and shallow body of water and exits through a rock fissure #10 to form a stream that exits by way of a swallet #27. The pool #18 is a very low velocity body of water fed from groundwater seepage. [file peerj-09-10757-s001.jpg]

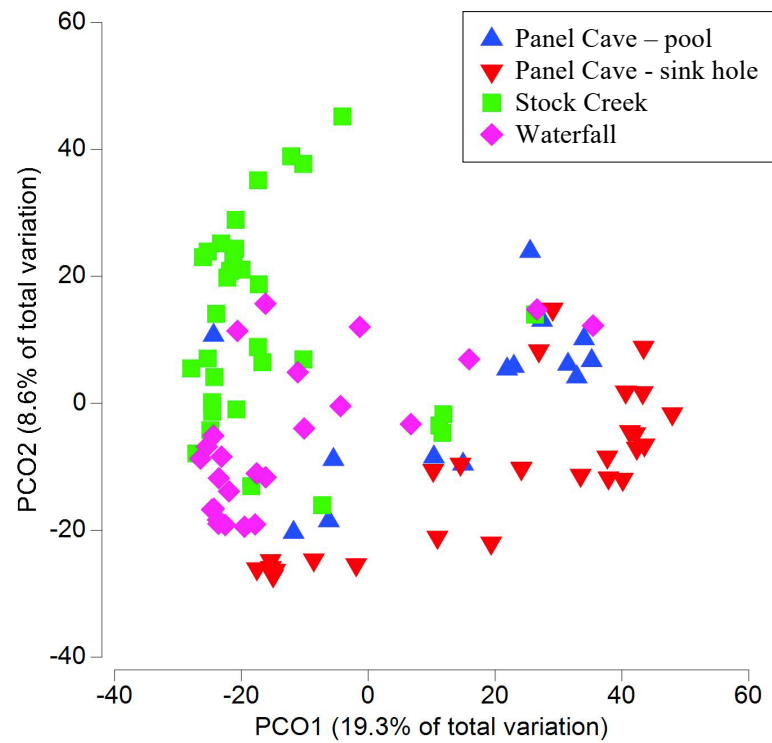

Supplemental Figure 2. PCoA plot of samples collected from Panel Cave and surface sites

Supplement: Supplemental Information 2 [file peerj-09-10757-s002.pdf]
