# Supplementary material for: Longitudinal metabarcode analysis of karst bacterioplankton microbiomes provide evidence of epikarst to cave transport and community succession
Source: PeerJ. 2021 Mar 8;9:e10757. doi: 10.7717/peerj.10757 (PMC7950216; doi:10.7717/peerj.10757)
Supplement: Supplemental Information 3 — (A) Bubble plot of the average ASV richness and the average total number of prokaryotic reads collected from each site at each timepoint. The y-axis represents the average ASV richness, bubble sizes represent the average number of reads, and the x-axis represents sample times. (B) The average calculated Shannon Indexes with standard errors for each for each site and time. [file peerj-09-10757-s003.pdf]

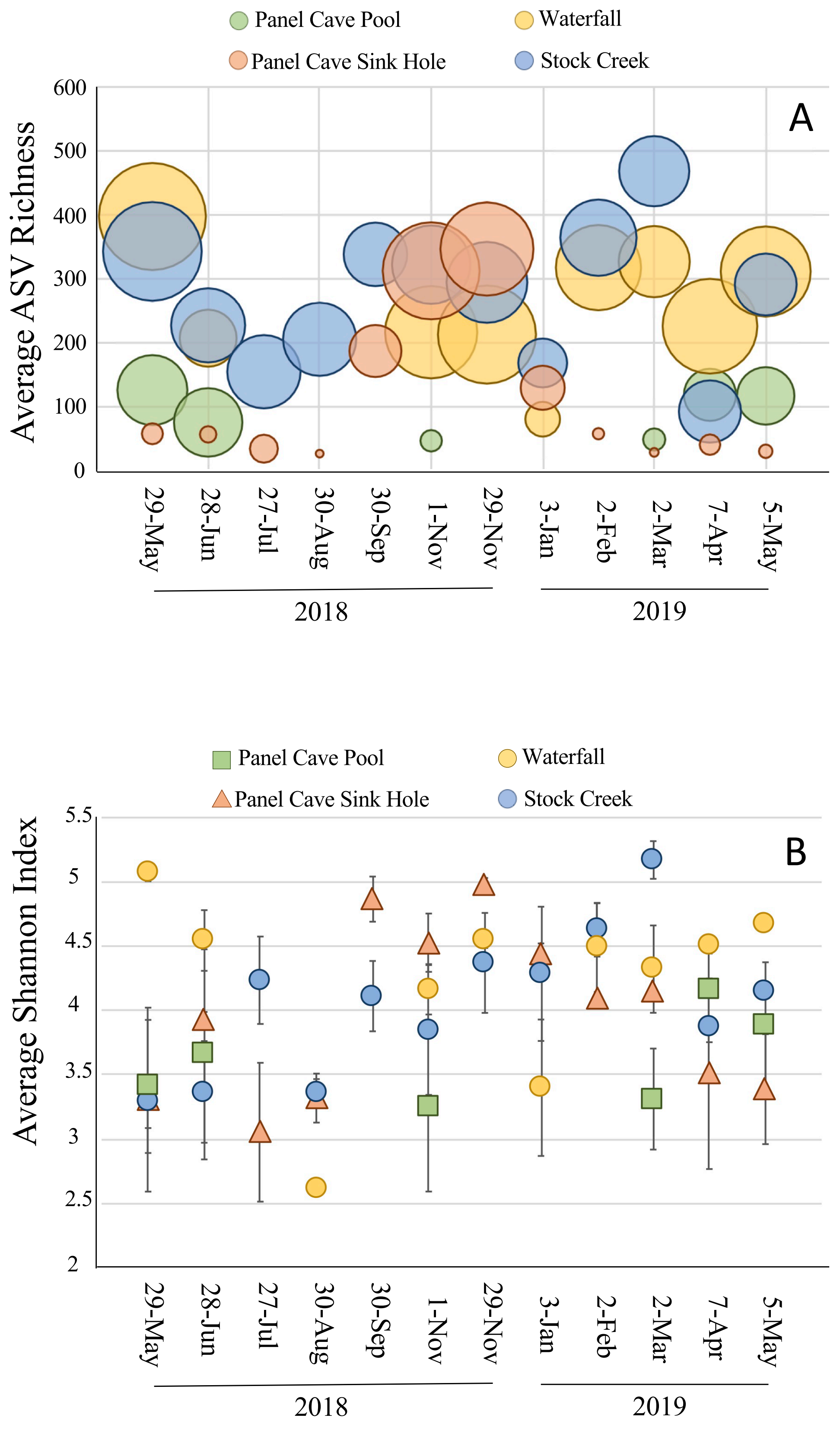

Supplemental Figure 3. (A) Bubble plot of the average ASV richness and the average total number of prokaryotic reads collected from each site at each timepoint. The y-axis represents the average ASV richness, bubble sizes represent the average number of reads, and the x-axis represents sample times. (B) The average calculated Shannon Indexes with standard errors for each for each site and time.
